# The complex metabolic network gearing the G_1_/S transition in leukemic stem cells: Hints to a rational use of antineoplastic agents

**DOI:** 10.18632/oncotarget.5155

**Published:** 2015-09-11

**Authors:** Theodora Stivarou, Maria Grazia Cipolleschi, Massimo D'Amico, Antonella Mannini, Enrico Mini, Elisabetta Rovida, Persio Dello Sbarba, Massimo Olivotto, Ilaria Marzi

**Affiliations:** ^1^ Department of Experimental and Clinical Biomedical Science, University of Florence, Florence, Italy; ^2^ DI.V.A.L. Toscana s.r.l., Florence, Italy; ^3^ Department of Experimental and Clinical Medicine, University of Florence, Florence, Italy; ^4^ Hellenic Pasteur Institute, Athens, Greece

**Keywords:** hypoxia, embryonic transcription factors, LY309887 and raltitrexed, folate metabolism, redox state

## Abstract

We defined the stem cell profile of K562 line, demonstrating the expression of the Embryonic Transcription Factors Oct3/4, Sox2, Klf4 and Nanog. This profile was associated with a high vulnerability to the physiological oxidizable substrate pyruvate. remarkably, this substrate was shown to be innocuous, even at the highest doses, to normal differentiated cells. This vulnerability is based on a complex metabolic trim centered on the cellular redox state expressed by the NADP/NADPH ratio geared by the mitochondrial respiratory chain. Flow cytometry revealed that the inhibition of this chain by antimycin A produced cell accumulation in the S phase of cell cycle and apoptosis. This block negatively interferes with the aerobic synthesis of purines, without affecting the anaerobic synthesis of pyrimidines. This imbalance was reproduced by using two antifolate agents, LY309887 and raltitrexed (TDX), inhibitors of purine or pyrimidine synthesis, respectively. All this revealed the apparent paradox that low doses of TDX stimulated, instead of inhibiting, leukemia cell growth. This paradox might have significant impact on therapy with regard to the effects of TDX during the intervals of administration, when the drug concentrations become so low as to promote maintenance of dormant cancer cells in hypoxic tissue niches.

## INTRODUCTION

Modern cancer chemotherapy include agents which interfere with intricate fundamental metabolic networks [1, 2]. The fact that these networks are often not thoroughly defined may make it difficult to predict the effects of a therapeutic strategy. A further difficulty derives from the necessity of exploring the effects of therapeutic agents in hypoxia, because cancer stem cells have a tendency to adapt to, and survive in, these environmental conditions [3, 4], implying high resistance to radio- and chemo-therapy [5]. Recently, crucial information in this field has been obtained with the identification of *embryonic stem cells* (ESC) [6] within the bulk of highly anaplastic tumors of any hystogenesis whatever [3, 4]. These cells can be identified from their expression of *embryonic transcription factors* (ETF), whose physiological role is the silencing of differentiation genes, and thus the maintenance of the undifferentiated embryonic state [7–11]. ESC originate at the gastrula stage under hypoxic conditions and migrate into adult tissues, becoming the *adult stem cells* after drastically reducing their self-renewal and plasticity. The ectopic finding of ESC in an adult tissue is now regarded as a potential warning that a cancer is about to begin or return, while the presence of ESC within a tumour is a crucial sign of severe anaplasia [12–14].

**Scheme 1:**
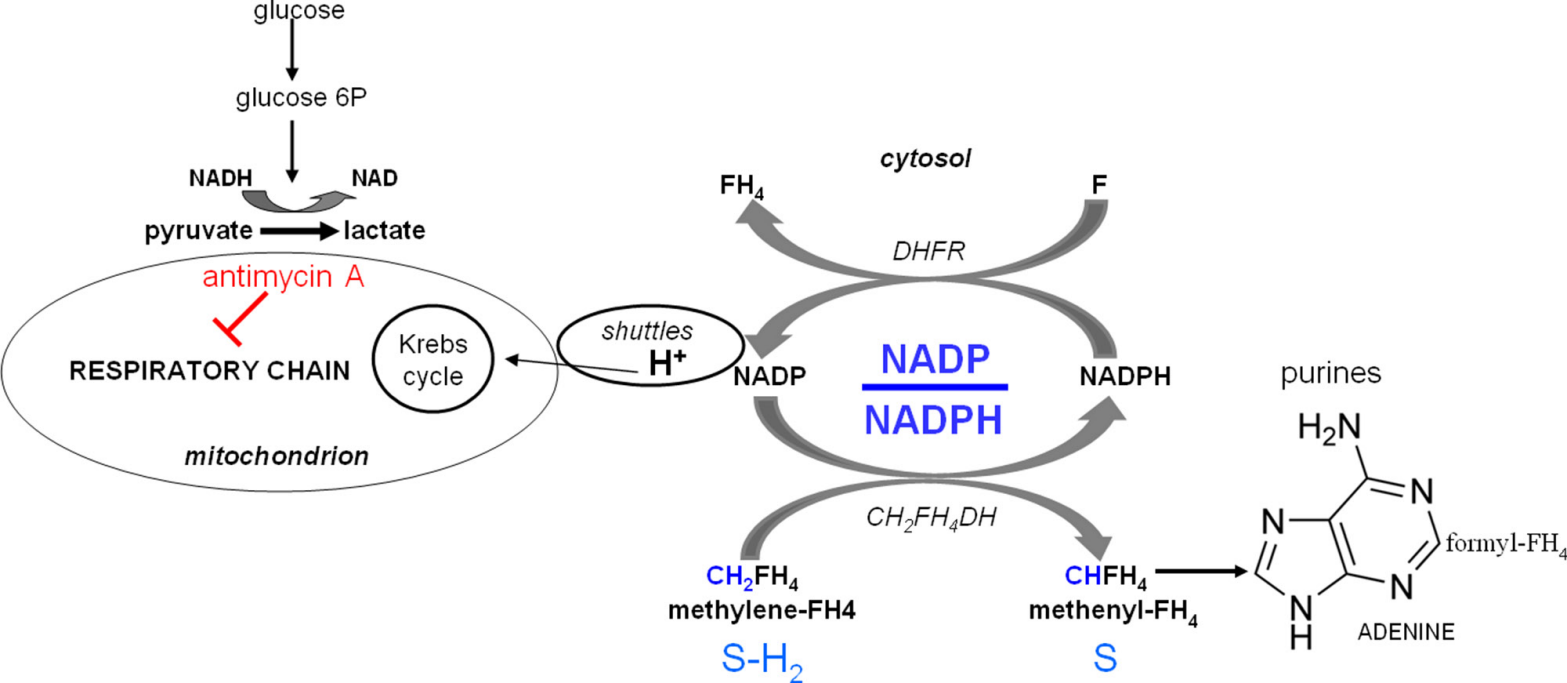
Role of the cellular redox state in the control of purine synthesis The core of this metabolic network is the cell redoxstate expressed by the cytosolic NADP/NADPH ratio. This ratio regulates the transfer of reducing equivalents (H^+^) from the methylen-tetrahydro-folate (CH_2_-FH_4_) to methenyl-tetrahydro-folate (CH-FH_4_). This NADP-dependent reaction (generating NADPH) is a limiting step of the synthesis of purine ring required for the amplification of purine pools indispensable for G_1_-S transition. A fundamental role in the regulation of NADP/NADPH ratio is played by folate, whose anaerobic reduction to FH_4_ by dehydrofolate-reductase (DHFR) generates NADP, compensating the anaerobic restriction of this factor. Whatever the mechanism increasing cytosolic NADPH, including pyruvate, glutamine through the glutaminolytic pathway, or the inhibition of DHFR by an excess of FH_4_, the cytosolic NADPH increase has an inhibitory effect on cell recruitment into S, unless it is removed by the shuttle mechanisms, which discharge the cytosolic reducing equivalents onto the mitochondrial respiratory chain. This role of the shuttles accounts for the fact that the activity of the chain is crucial for the G_1_/S transition, which is impaired by a specific inhibitor like antimycin A or by anaerobiosis. A similar inhibition can be brought about out in air whenever the chain, although not inhibited, is saturated by reducing equivalents produced by oxidizable substrates of the Krebs cycle, mostly pyruvate. This inhibitory effect of pyruvate accounts for the crucial role of its disposal of by the reduction to lactate, and the consequent exportation into the environment, in hypoxia-adapted cancer cells in air (the aerobic glycolysis, traditionally named the Warburg's effect).

Metabolic studies [3, 4, 15] carried out in our laboratory employing the highly anaplastic Yoshida's ascites hepatoma AH130 cells revealed the basis of cancer adaptation to hypoxia. We demonstrated that the very same metabolic features responsible for this process are accompanied by the susceptibility to a powerful cytotoxic effect exploited by physiological factors, such as pyruvate, tetrahydrofolate (FH_4_) and glutamine. This paradox is also found in human anaplastic tumors with a totally different histogenesis, and reflects a complex metabolic interference of these agents with purine metabolism. This interference culminates in the impairment of the amplification of the purine base pools which is required to fulfil the G_1_/S transition of cell cycle. Such an impairment implies an inadequate mitochondrial apparatus, like that characterizing the hypoxia-adapted cells surviving in the dormant state by means of a glycolysis-oriented metabolism [15, 16]. This trim was first discovered by Warburg in 1923 in cancer cells, describing the *aerobic glycolysis*: this metabolic pathway consists of the arrest of aerobic utilization of glucose at the level of pyruvate, which is mostly exported upon reduction to lactate [15, 17, 18]. This is a tremendous waste of the energy which could be produced by the aerobic degradation of glucose to H_2_O and CO_2_ through the Krebs cycle and the mitochondrial respiratory chain. We demonstrated that in this metabolic configuration the addition of an excess of pyruvate will lead to the saturation of respiratory chain at the expense of the re-oxidation of crucial cytosolic dehydrogenation steps geared by the NADP/NADPH ratio [3, 4]. Indeed, the reducing equivalent (H^+^ or electrons) derived from these dehydrogenations cannot be disposed of through the metabolic shuttles [19] leading to the respiratory chain (see Scheme 1). This sequence will inhibit the growth of any type of anaplastic cancer, through a ten-fold reduction of the cellular NADP/NADPH ratio, that is, at the same level in anaerobiosis as in the presence of antimycin A, a powerful specific inhibitor of the respiratory chain [4, 15]. The pyruvate inhibition is removed by the addition of preformed purine bases (adenine or guanine) as well as by folate (F), indicating that its mechanism is mediated by the impairment of the NADP-dependent dehydrogenation of methylen-FH_4_ (CH_2_-FH_4_) to methenyl-FH_4_ (CH-FH_4_), catalyzed by the CH_2_-FH_4_-dehydrogenase [3, 4, 19].

In the present study, we tested the antitumor activity of pyruvate on Chronic Myeloid Leukemia (CML) cells of the K562 stabilized line [20]. A high percentage of these cells expresses ETF and exhibited a high sensitivity to pyruvate. Thus, K562 cells turned out to be suitable for establishing whether and how much CML is subjected to the same metabolic network previously described in Yoshida's hepatoma cells [3, 4]. We answered to these questions positively, taking advantage of two antifolate selective inhibitors of the synthesis of purine or pyrimidine DNA precursors, such as LY309887 [21, 22] and raltitrexed (TDX) [22], respectively. These compounds display potent activity against human leukemia cells [23–26], and may turn out to be effective agents for the clinical treatment of leukemia [27]. LY309887 is a folate analogue that selectively inhibits glycinamide-ribonucleotide-formyl-transferase (GARFT) [28], while TDX selectively targets thymidylate-synthase (TS) [29]. Our experiments revealed that the reductive shift of the hypoxic NADP/NADPH ratio determines the imbalance of the purine/pyrimidine ratio, altering the correct base assembly into DNA by DNA-polymerase. These results made it possible to observe and explain the apparent paradox that, in hypoxic conditions, low concentrations of TDX stimulate, instead of inhibiting, K562 cell growth.

## RESULTS

### The pluripotent stem cell profile of the K562 cell line: ETF expression

**Figure 1:**
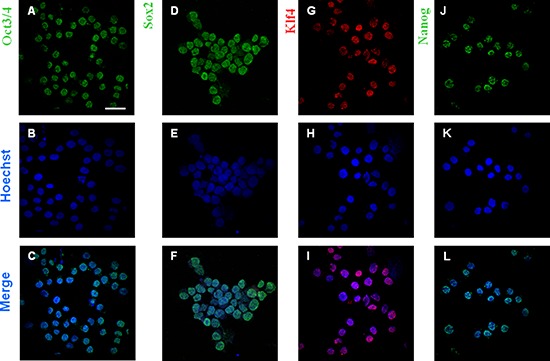
Expression of ETF in K562 cells ETF (Oct3/4, Sox2, Klf4, Nanog) expression in K562 cells. Bar: 50 μm; magnification: 60x. Cell nuclei were stained with Hoechst 33258.

Fig. 1 shows that up to 60–70% of the K562 cell population expresses four fundamental ETF, such as Oct3/4, Sox2, Klf4 and Nanog. These ETF are so crucial for the maintenance of the ESC undifferentiated phenotype that their transfection is sufficient *to reprogram* adult differentiated cells, of any histogenesis whatever, down to the pluripotent state [8, 9]. We previously found that ETF expression in tumors is associated with vulnerability to pyruvate, a phenomenon which represents the hallmark of the adaptation of ESC to hypoxia, and that the detrimental effect of pyruvate on cell number in culture increases in proportion to the level of tumor anaplasia, being maximal for AH130 cells [3, 4]. The underlying metabolic scenario was deepened as described hereunder.

### K562 cell growth inhibition by pyruvate or in hypoxia and the effects of folate addition

**Figure 2:**
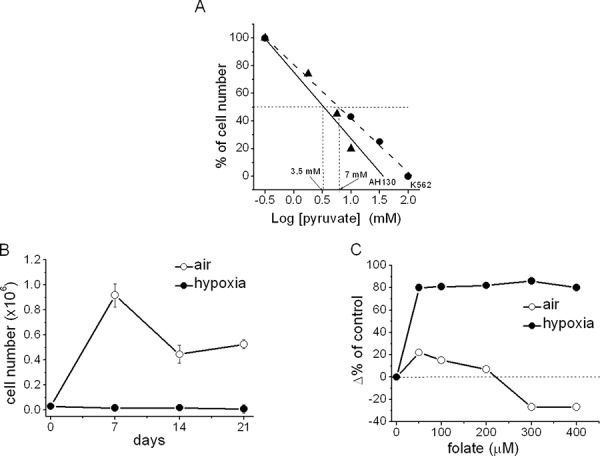
The sensitivity to pyruvate of K562 cells in comparison with AH130 cells. Hypoxic inhibition of K562 cell growth and its removal by folate addition **A.** concentration-dependence of the detrimental effect of pyruvate on viable cell number in culture. Data are expressed as percent variation with respect to control values at day 7 of incubation in air. Triangles: AH130 cells; circles: K562 cells. **B.** Cells were incubated in air (open circles) or hypoxia (0.1% O_2_; solid circles) for the indicated times. Values (total number of viable cells) are means ± S.E.M. of three independent experiments. **C.** Cells were incubated for 21 days in air (open circles) or hypoxia (solid circles) in the presence of different folate concentrations. Data are expressed as percent variation (Δ%) with respect to the control value (day-21 cultures).

Fig. 2A shows that pyruvate concentration-dependently reduced K562 cell number in day-7 cultures incubated in air. Thus, K562 cells seem sensitive to pyruvate similarly to AH130 cells. This similarity allows allows to evaluate K562 cells with respect to the metabolic analysis performed on AH130 cells. As summarized in the Introduction, the pyruvate inhibition of cell growth is removed by the addition of purine bases or folate to cultures. On this basis, we determined the effects of folate on the growth kinetics of K562 cells incubated in air or severe hypoxia (0.1% O_2_ in the incubation atmosphere). In air, the cell population grew up to 9 × 10^5^ cells/ml at day 7 and declined thereafter to reach a steady-state at about 4–5 × 10^5^ cells/ml at days 14–21 (Fig. 2B). This decline is due to cell crowding, with the consequent nutrient shortage in the absence of medium renewal. When cultures were incubated in hypoxia, cell growth was abolished [30–34]. Fig. 2C shows that, at day 21 of incubation in hypoxia but not in air, folate addition markedly increased the total cell number in comparison to the value obtained for the respective untreated control. It is worth noting that relatively low concentrations of folate determined a maximal increase of cell number in hypoxia-incubated cultures.

### Effects of antimycin A on the cytosolic NADP/NADPH ratio in K562 cells

**Figure 3:**
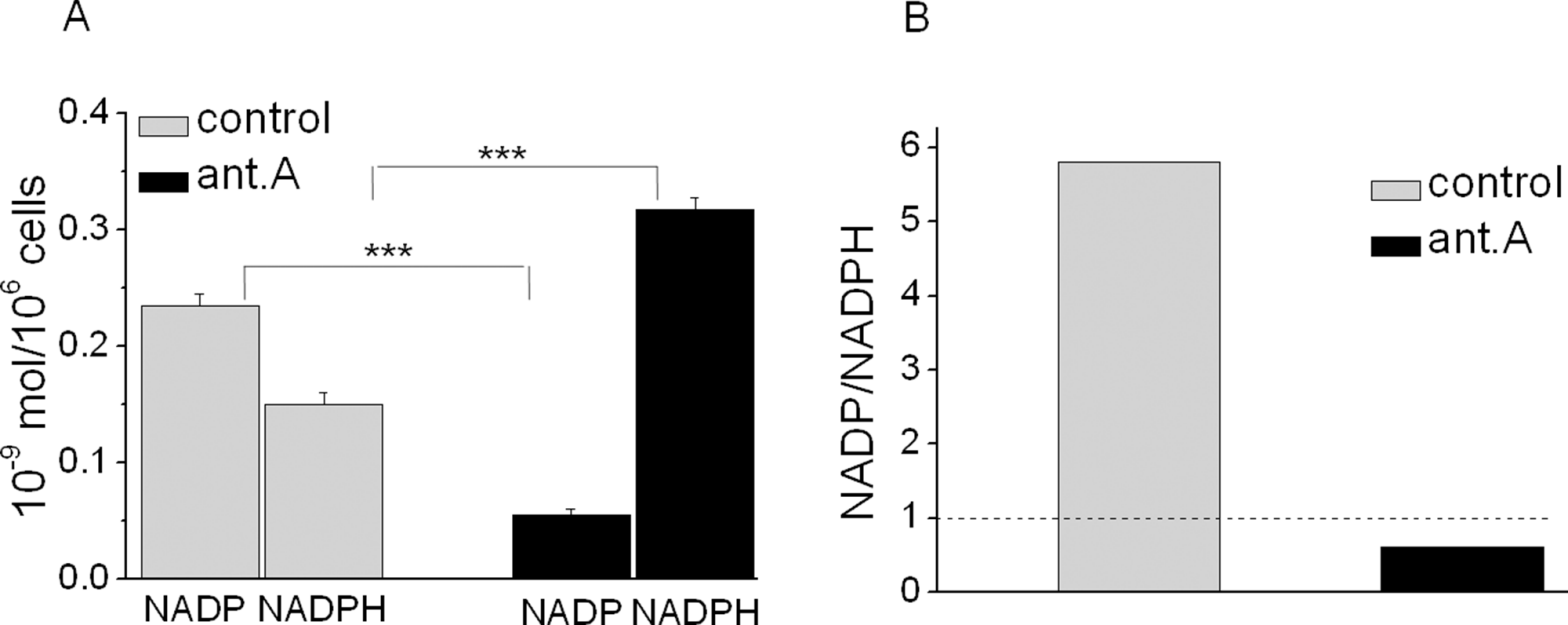
Effects of antimycin A on NADP and NADPH levels and their ratio in K562 cells Cells were incubated for 4 days in air in the presence or the absence of 6 × 10^−6^M antimycin A and metabolite concentrations measured by HPLC technique. **A.** values, expressed as nmol ×10^6^ cells, are means ± S.E.M. of data obtained in triplicate. **B.** NADP/NADPH ratios from data reported in panel A.

Fig. 3 shows that the treatment with antimycin A (6 × 10^−6^M), an inhibitor of complex III of mitochondrial respiratory chain, of cultures incubated in air for 4 days produced a drastic reductive shift (Fig. 3A), resulting in a marked decrease of the NADP/NADPH ratio (Fig. 3B). We had previously shown that this shift is abolished by folate addition [4] through the reaction F + NADPH → FH_4 +_ NADP. Provided folate is abundant, this anaerobic reaction supplies the NADP necessary for the activity of cytosolic dehydrogenases, including CH_2_-FH_4_-dehydrogenase, which allows purine synthesis in hypoxia [4] (see Scheme 1). Altogether, these data suggested that K562 cell recruitment into the cycling state is geared by the NADP/NADPH ratio as a function of respiratory chain activity or folate supply.

### Effects of antimycin A on total number and cell cycle distribution of K562 cells

**Figure 4:**
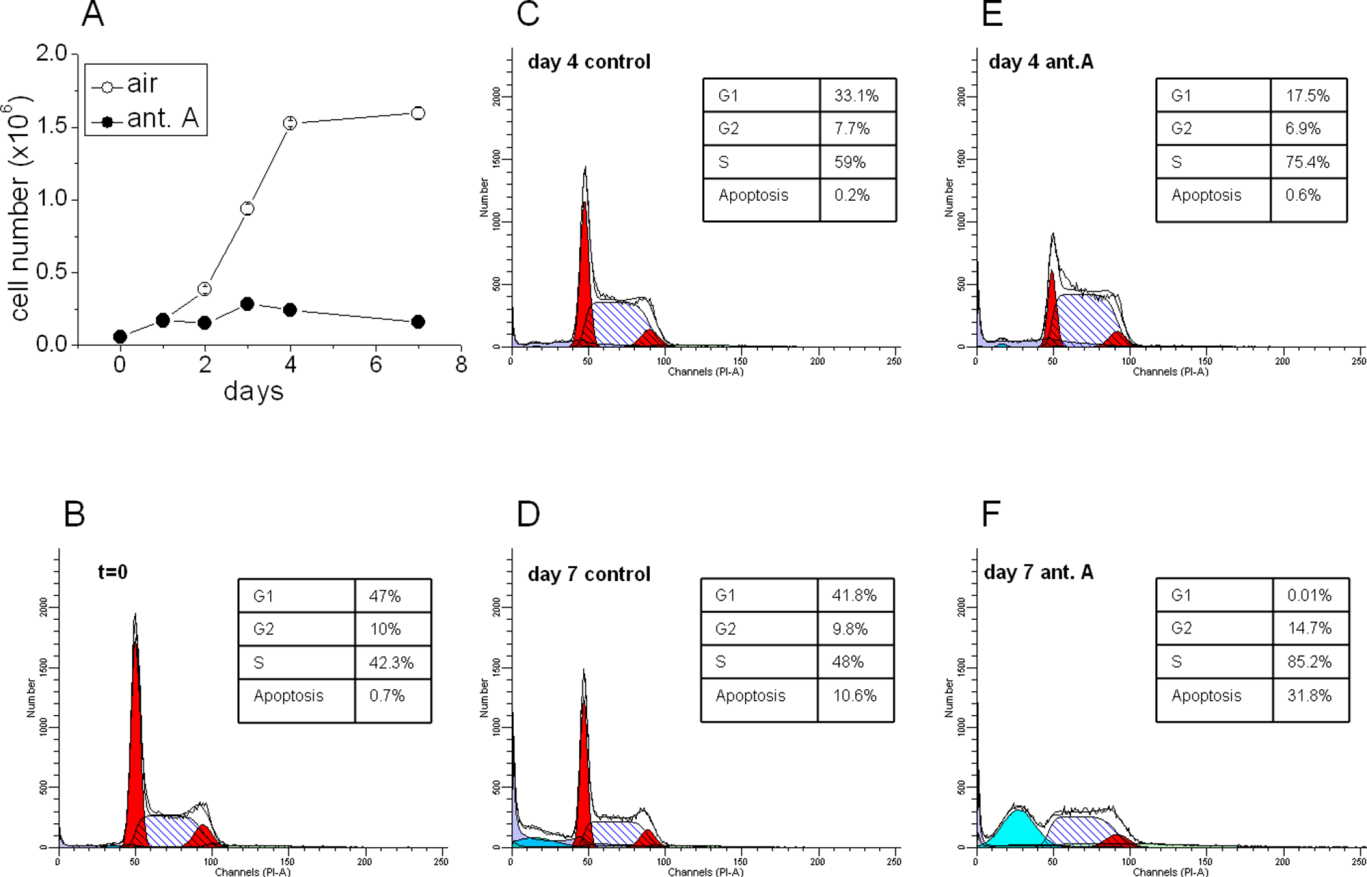
Effects of antimycin A on K562 cell growth and cell cycle distribution **A.** Cells were incubated for the indicated times in air (open circles) or in the presence of 6 × 10^−6^M antimycin A (solid circles). Values (total number of viable cells) are means of ± S.E.M. of data from three independent experiments. **B–F.** Cell cycle distribution, measured by flow cytometry, in air (control) versus antimycin A (ant.A) at the indicated incubation times.

Fig. 4A shows that cell growth occurring in air was markedly inhibited following antimycin A addition. This inhibition was apparently independent of the stabilization of Hypoxia-Inducible Factor (HIF), which is driven via the activation of specific sensors of low oxygen tension [35]. The effects of antimycin A on cell cycle, as determined by flow cytometry, are shown in Fig. 4B–4F. In control cultures, when cell population attains the steady state (C, D), cell percentage progressively increases in the G_1_ phase of cell cycle and decreases in S, undergoing evident apoptosis at day 7 (D). In antimycin A-treated cultures, a marked cell accumulation in S was already evident at day 4 (E), followed by a further increase and massive apoptosis at day 7 (F). Thus, the antimycin A block of respiratory chain interfered with cell transition through cell cycle. These data are very similar to those reported by Hastak [36], showing that the imbalance of pyrimidine pools by *N*-(phosphonacetyl)- *L*-aspartate (PALA) produces base misincorporation into DNA during the first S phase, which is sufficient to bring about cell death during the second cell cycle.

### Effects of purine synthesis inhibition and adenine on the total number and cell cycle distribution of K562 cells

**Figure 5:**
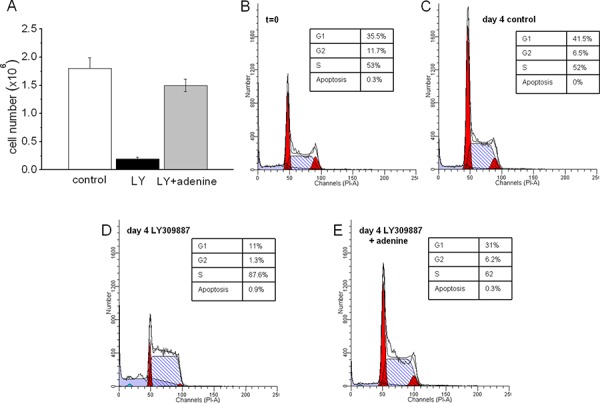
Effects of inhibition of purine synthesis and its removal by adenine K562 cells were incubated in air for 7 days (A) or for the indicated times (B–E) in the absence (control) or the presence of 10^−9^M LY309887 (LY) or LY plus 0.1 mM adenine (LY+ad). **A.** values represent the total number of viable cells at day 7 of incubation and are means ± S.E.M. of three independent experiments. **B–E.** cell cycle distribution at the indicated incubation times in cultures incubated in the absence (control) or the presence of LY309887 or LY309887 + adenine.

LY309887, a specific inhibitor of purine synthesis, markedly reduced the total number of cells at day 7 of incubation in air (Fig. 5A), well in keeping with the effects of antimycin A shown above. Such a reduction was abolished by the addition of adenine to LY309887-treated cultures. These effects are also evident from Fig. 5B–5E, which shows that the cell cycle distribution changes induced by the inhibitor at day 4 (D *vs* C) were abolished in the presence of adenine (E). This effect implies that the endogenous pyrimidine pool is *per se* able to sustain K562 cell growth, once the insufficient endogenous purine pool is amplified by the addition of adenine.

### Effects of antimycin A on the A/T and G/C ratios

**Figure 6:**
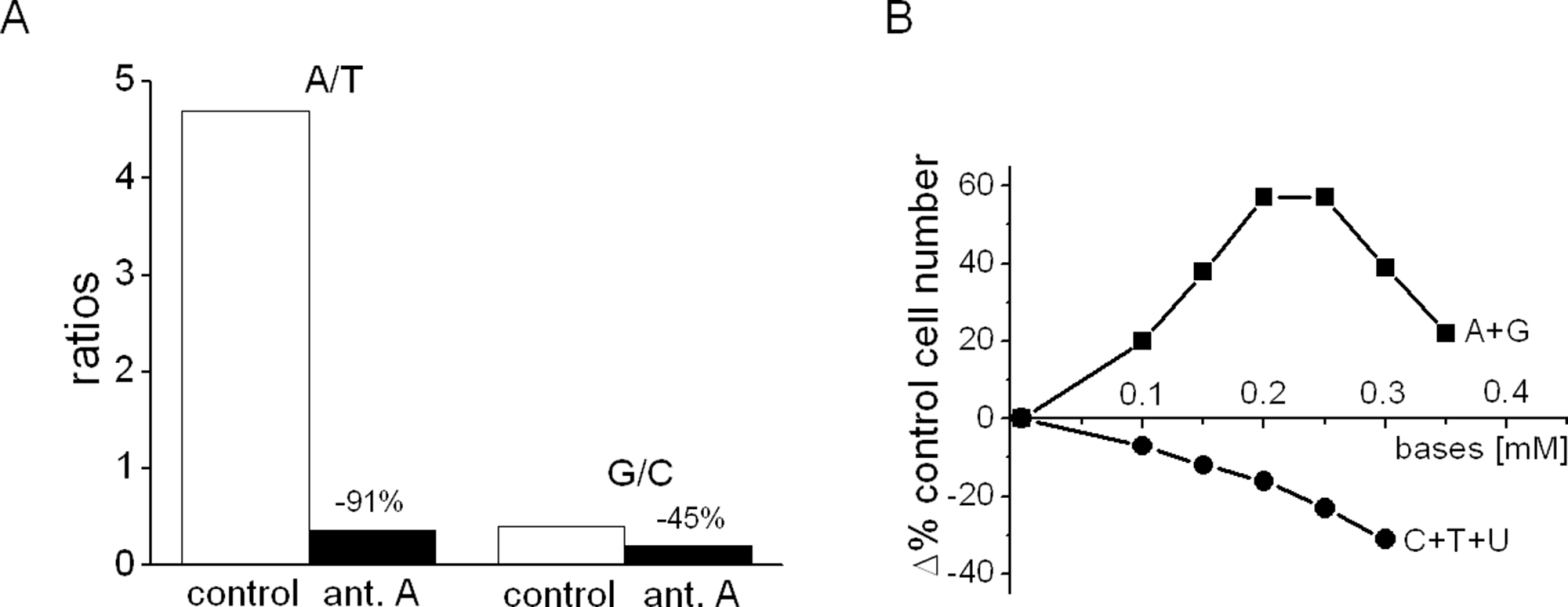
Effects of antimycin A on the A/T and G/C ratios in AH130 cells and of purine or pyrimidine addition on K562 cell number **A.** AH130 cells were incubated for 18 h in air in the absence (control) or the presence of 6 × 10^−6^M antimycin A (ant.A) and base concentrations determined by HPLC technique; A/T = adenine/thymine, G/C = guanine/cytosine ratios. **B.** K562 cells were incubated for 7 days in the presence of 6 × 10^−6^M antimycin A and in the absence or the presence (from time zero) of different concentrations of purines (A + G) or pyrimidines (C + T + U) at the 1:1 ratio. Values represent the total number of viable cells at day 7 of incubation and are expressed as percent variation (Δ%) with respect to the value obtained for the base-untreated control (antimycin A alone).

The above data must be interpreted in the light of the available information about the close association between DNA synthesis and turnover and the balance of various deoxy-nucleotide-triphosphate (dNTP) pools [36]. It is because of this close association that any imbalance between purines and pyrimidines will produce a high ratio of base misincorporation of these precursors into DNA, generating mutations. Up to a certain limit, these mutations can be repaired by an efficient p53, whereas they induce cell apoptosis when the DNA “house keeper” is deleted. We found that p53 is totally deleted in K562 as well as AH130 cells [3], implying that in these cells any serious alteration of the purine/pyrimidine ratio, such as that caused by a restricted purine synthesis or the impairment of respiratory chain by pyruvate or antimycin A [3, 4, 15], will promote lethal apoptosis. Consistently, as shown in Fig. 6A, antimycin A brought about a 14-fold decrease of the adenine/thymine ratio (A/T) in AH130 cells, whereas it only halved the guanine/cytosine ratio (G/C). This indicates that antimycin A determined a major effect on the purine pool (adenine and guanine), with a minor effect on the pyrimidine pool (thymine and cytosine). This conclusion was confirmed by the experiments reported in Fig. 6B, which shows the concentration/response relationship of the artificial amplification (at time 0 of incubation) of the purine or pyrimidine pools to K562 cell number (at day 7) in the presence of antimycin A. Purines determined an increase of cell number by up to almost 60%, while pyrimidines produced only negative effects. Purine addition determined an increase up to 0.25 mM and a marked decline at higher concentrations. Pyrimidine addition determined a decrease of K562 cell number over the whole range of concentrations (0–0.30 mM).

These results led us to draw the following inferences: a) the purine pools are made deficient by the block of respiratory chain (antimycin A), accounting for the reduction of cell number in culture with respect to control; this reduction is reverted by base addition; beyond a well-defined limit, the increase of purine pools becomes detrimental, despite a good adenine/guanine balance; this limit is evidently attained when the ratio purine/pyrimidine is optimal; b) the pyrimidine pools are optimal, or at least sufficient, for maximal growth in the presence of antimycin A, so that their further amplification is only detrimental, even at minimal concentrations. Thus, in summary, the optimal growth conditions for K562 cells in air are guaranteed by a well-defined purine/pyrimidine ratio; this equilibrium is altered by hypoxia, where the oxygen-dependent purine synthesis is depressed, leaving the pyrimidine pools unaltered.

### Effects of purine or pyrimidine synthesis inhibition in air in the absence or the presence of antimycin A

**Figure 7:**
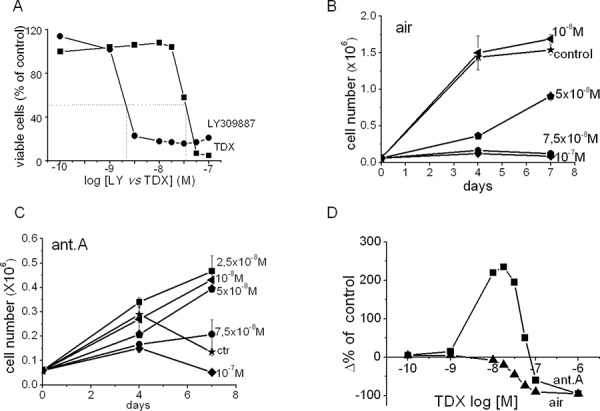
Effects of the inhibition of purine or pyrimidine synthesis in air in the presence or the absence of antimycin A on the time-course of K562 cell number in culture. The raltitrexed paradox **A.** Cells were incubated in air in the absence or the presence of the indicated concentrations of raltitrexed (TDX; squares) or LY309887 (circles) and the number of viable cells in culture counted at day 7 of incubation. Data are expressed as % of values obtained for the drug-free control. **B, C.** Cultures were incubated in air in the absence (air) or the presence (ant.A) of antimycin A and in the absence (control) or the presence of the indicated concentrations of TDX, added at time 0; the total number of viable cells in culture was counted at the indicated times of incubation. Values are means ± S.E.M. of three independent experiments. **D.** Cells were incubated in air in the absence (air; triangles) or the presence (ant.A; squares) of antimycin A and in the presence of the indicated concentrations of TDX; the total number of viable cells in culture was counted at day 7 of incubation. Data (derived from panels B and C) are expressed as Δ% of TDX-free control.

The above data and inferences suggested to us to compare the effects of specific inhibitors of purine or pyrimidine synthesis, such as LY309887 or raltitrexed (TDX), respectively, on the recruitment of K562 cells to growth. Fig. 7A shows the concentration/response effects of LY309887 or TDX on the total number of viable K562 cells in cultures incubated in air for 7 days. LY309887 started to inhibit cell growth at 10^−9^ M, reaching the maximal effect around 5 × 10^−8^M; this indicates an extreme sensitivity of purine pools to this inhibitor. On the other hand, the inhibition brought about by TDX started only at 5 × 10^−8^ M, to reach the maximum at almost 10^−7^M, indicating that K562 cells are at least ten times more sensitive to the restriction of purines than to that of pyrimidines. In other words, the endogenous purine pool of these cells in air is insufficient to bring about cell cycling upon exposure to even minimal concentrations of LY309887. By contrast, the pyrimidine pool remains sufficient for the maximal growth in the presence of relatively high concentrations of TDX.

The crucial role played by the balance of DNA base pools emerged from the experiments reported in Fig. 7B–7D, where the concentration/response effects of TDX on viable cell number in the absence or the presence of antimycin A were compared. Fig. 7B, 7C shows the effects of TDX on the time-course of K562 cell number in cultures incubated in air in the absence (B) or the presence (C) of antimycin A. TDX concentration-dependently inhibited cell growth in air, whereas, in the presence of antimycin A, TDX produced an unexpected stimulation of cell growth in the range 10^−8^ to 5 × 10^−8^M. This counterintuitive result indicates that a modest inhibition of pyrimidine pool by low concentrations of TDX stimulated cell growth by attenuating the marked disequilibrium between the purine and pyrimidine pools. This phenomenon is even more clearly shown in Fig. 7D, where the data relative to the effects of TDX on viable cell number at day 7 of incubation are reported as percentages of the relative control (TDX-untreated, incubated in the presence or the absence of antimycin A). Such a paradox makes it predictable that TDX could have a detrimental impact on therapy, promoting the growth resumption of dormant cancer stem cells hidden in hypoxic niches [37] (see Discussion).

## DISCUSSON

The results of this study led us to conclude that K562 cells are a CML cell population endowed with an epigenetic and metabolic trim that parallels the neoplastic progression of a number of malignant tumors of different histogenesis. This trim characterizes the *tumor converging phenotype* [15], whose essential feature is an extreme morphological and metabolic simplification centered on a high nucleus/cytoplasma ratio and a poor, easily-saturated, mitochondrial apparatus. This feature, which may well be responsible for cancer radio- and chemio-resistance, resembles the one we described for ESC expressing ETF [38, 39], whose energetic metabolism, based on aerobic glycolysis, is vulnerable to pyruvate, as outlined in the Introduction. We showed that this metabolic configuration predisposes to unbalance the purine/pyrimidine ratio, leading to cell cycle arrest and apoptosis [36], a tendency that is realized in hypoxia.

**Scheme 2:**
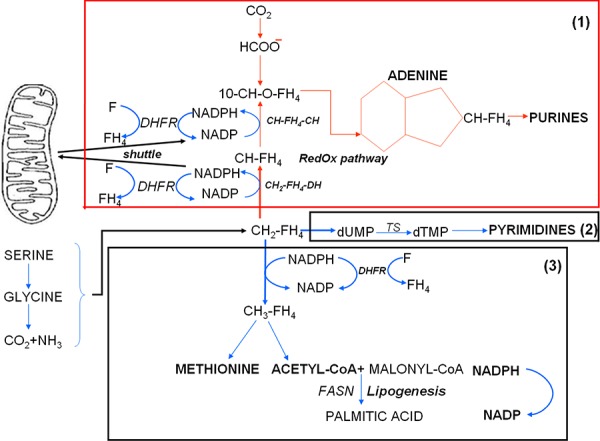
The metabolic network controlling the synthesis of DNA bases as a function of folate metabolism See Scheme 1 and its legend as well as the Discussion. CH-FH_4_-CH: CH-FH_4_-cyclohydrolase; TS: Thymidilate Synthase.

Scheme 2 shows the biochemical pathways implicated in DNA base synthesis [40], which share CH_2_-FH_4_ as the initial metabolite and set in motion no less than three syntheses, those of: (1) purines; (2) pyrimidines; (3) methionine and acetylCoA derivatives of methyl-FH_4_ (CH_3_-FH_4_). Pathway (3) leads to the anaerobic synthesis of CH_3_-FH_4_ through the reduction of CH_2_-FH_4_ by the NADPH-dependent CH_2_-FH_4_-reductase, producing NADP. Pathway (2) consists of the anaerobic conversion of dUMP to dTMP catalysed by thymidylate-synthase (TS), in which CH_2_FH_4_ acts as a co-substrate, one-carbon unit donor. Pathway (1) generates the purine pools through the *RedOx pathway,* so-called to indicate its dependence on the NADP/NADPH conversion [40]: this pathway, in fact, requires NADP at two crucial steps: a) the oxidation of CH_2_-FH_4_ to CH-FH_4_ catalyzed by the CH_2_-FH_4_-dehydrogenase; b) the conversion of CH-FH_4_ to formyl-FH_4_ (10-HCO-FH_4_) catalyzed by the CH-FH_4_-cyclohydrolase. Both a) and b) reactions are strictly NADP-dependent, requiring a high NADP/NADPH ratio, so that they are inhibited when the respiratory chain is blocked by antimycin A or saturated by pyruvate. This inhibition can be removed by the addition of folate through the reaction F + NADPH → FH_4_ + NADP catalyzed by the dihydrofolate-reductase (DHFR). Thus, it seems straightforward to assume that hypoxia or antimycin A block purine synthesis, while they favour pyrimidine synthesis via the maintenance of CH_2_FH_4_ levels. On this assumption, one would expect either treatment to generate an imbalance between the two types of bases, altering the DNA synthesis operated by DNA-polymerase, which requires a strict equilibration of their ratio. More important still, we were able to show that, in the absence of this equilibrium, a high level of misincorporations occurs, likely to generate mutation and hence apoptotic cell death [36, 41].

With regard to cancer chemotherapy, the biochemical network summarized in Scheme 2 needs to be better characterized. In this respect, the K562 cell line emerged as a suitable model to study the response of blast-crisis CML cells to “antifolate” drugs such as LY309887 or TDX. This biochemical network, on the other hand, is of general relevance to determine the role of cells expressing ETF in the onset and development of cancer. In this respect, it is worth pointing out that ESC are physiologically generated in the gastrula, an early embryo stage characterized by hypoxia. Indeed, the occurrence of ESC-like cells in the tumor bulk is generally supposed to derive from their capacity to home in hypoxic tissue niches, where they can survive indefinitely in a dormant state [37]. These cells can be generated in the bulk of tumors through the epigenetic reprogramming of adult cells triggered and sustained in the course of neoplastic progression [13], which indeed consists of an uncontrolled drift to DNA mutations called chromatin plasticity. Yamanaka and Hochedlinger demonstrated that the absence of efficient p53 leads to chromatin instability, that strongly facilitates epigenetic reprogramming down to ESC [41–45]. Thus, the expression of ETF in the bulk of tumors, whatever their origin, represents a powerful drive to progression to an anaplastic phenotype. This evolution is the main factor determining the risk of relapses, which are impossible to prevent by the traditional antineoplastic treatments. At the same time, however, the ESC-like cells might consent a selective antitumor therapy based on their specific vulnerability to pyruvate, FH_4_ and glutamine, for which we here proposed the acronym of CCPF (Cancer- Cytotoxic Physiological Factors). According to our studies, via a strong reductive shift of NADP/NADPH ratio, CCPF impair the aerobic purine synthesis through the aerobic utilization of CH_2_-FH_4_ in the assembly of the 10-HCO-FH_4_ in the purine ring (pathway (1) of Scheme 2). The extraordinary advantage of this approach lies in the fact that CCPF, being physiological factors, are devoid of any general toxicity for human beings.

In the light of all above, the results presented in this paper are relevant to cancer therapy. Indeed, LY309887 or TDX would be able to kill the bulk of the tumor but not to interfere with the ETF-expressing stem cell compartment. Furthermore, the use of inhibitors point of pyrimidine synthesis, such as TDX, might generate the paradoxical effect demonstrated in this paper, namely, the restoration within the hypoxic stem cell niches of a more favourable equilibrium between the two types of DNA bases, thereby favouring the recruitment of dormant cells into the cycling state. The low inhibitor concentrations driving this effect correspond to the plasmatic levels which are usually reached at the end of each treatment cycle and during the necessary therapeutic intervals. This may represent a serious limit to the use of TS inhibitors On the other hand, it is also worth to be taken into consideration the possibility that the paradoxical effect of TS inhibitors turn out useful to rescue cell sensitivity to anti-proliferative agents if one were to adopt a combination therapy protocol.

## MATERIALS AND METHODS

### Cells and culture conditions

K562 cells were cultured in 24-well dishes (EuroClone) at 3 × 10^4^ /ml (2 ml/well) in RPMI 1640 medium supplemented with 50 units/ml penicillin, 50 μg/ml streptomycin, and 10% fetal bovine serum (all from EuroClone, Paignton, U.K., http://www.euroclone.net/).

Experiments were established with cells rescued from maintenance cultures, plated at 5 × 105/ml and incubated for 24 hours ("intermediate" passage) at 37°C in a water-saturated atmosphere containing 5% CO_2_ and 95% air, before the final replating into 24-well dishes. Incubation in severe hypoxia was carried out in a water-saturated atmosphere containing 0.1% O_2_, 5% CO_2_, 95% N_2_, in a gas-tight incubator/manipulator (Ruskinn Concept 400 or Don Whitley Scientific DG250 anaerobic workstation) flushed with the above preformed gas mixture. Such a workstation allows easy entry and exit of materials and sample manipulation without compromising the hypoxic environment. Controls of experiments in hypoxia have been carried out incubating cells in air, which is considered a standard condition for cell culture. However, it is worth pointing out that incubation in air generates an artificial hyperoxic condition which has nothing to do with the real situation occurring in even the best-oxygenated tissue environment [46].

Yoshida's ascites hepatoma AH130 cells were maintained and cultured as previously described [3].

Cell viability was assessed by trypan blue exclusion, diluting cell suspension 1:1 with a 1% wt/vol trypan blue solution (Sigma-Aldrich). The number of total and viable cells was counted in a Bürker hemocytometer.

### Immunofluorescence

Cell suspension was cytocentrifuged at 800 rpm for 15 min and cells processed essentially as previously described [3] and assayed with the following antibodies: rabbit anti-Nanog, 1:800; mouse anti-Klf4, 1:800; rabbit anti-Sox2, 1:800; rabbit anti-Oct3/4, 1:1000 (Stemgent). Cells were then incubated with Cy3-conjugated anti-mouse, or fluorescein-conjugated anti-rabbit secondary antibody (Chemicon) at 1:800 dilution. Cell nuclei were stained with Hoechst 33258, 1:2000 (Sigma-Aldrich).

### HPLC

Organic extracts were obtained from 10^7^ cells with 75% ultrapure acetonitrile plus 25% 10 mM KH_2_PO_4_ at pH 7.4. The extracts were completely dehydrated and suspended in 20 μl of running buffer and analyzed in a Perkin Helmer Series 200, UV/VIS Detector HPLC apparatus, as previously described [4].

### Flow cytometry

To determine cell cycle distribution, 5 × 10^5^ cells were centrifuged for 6 min at 1,200 rpm, resuspended in 500 μl of propidium iodide (PI) solution (trisodium citrate 0.1% w/v, NP40 0.1% w/v, PI 50 μg/ml) (Merck, Biosciences, Calbiochem respectively) and incubated for 30 min at 4°C in darkness. Cells were then subjected to flow cytometry (Becton-Dickinson FACS-Canto) and data analyzed, to determine cell cycle distribution, by the Mod fit LT software [3].

### Drugs and chemicals

Pyruvate, antimycin A, folic acid, purines and pyrimidines were purchased from Sigma Aldrich and added at the concentration indicated in the legends. Antimycin A was dissolved in ethanol.

Raltitrexed (TDX) and LY309887 were kindly supplied by Prof. Enrico Mini, Department of Experimental and Clinical Medicine, University of Florence, Italy.

### Statistical analysis

Significance levels were calculated by the Student's *t*-test: * = *p* < 0.05; ** = *p* < 0.02; *** = ***p*** < 0.005.
